# Beta-blockers in cardiac arrhythmias–Clinical pharmacologist’s point of view

**DOI:** 10.3389/fphar.2022.1043714

**Published:** 2023-01-09

**Authors:** Łukasz Wołowiec, Grzegorz Grześk, Joanna Osiak, Aleksandra Wijata, Martyna Mędlewska, Patryk Gaborek, Joanna Banach, Anna Wołowiec, Mariola Głowacka

**Affiliations:** ^1^ Department of Cardiology and Clinical Pharmacology, Faculty of Health Sciences, Collegium Medicum in Bydgoszcz, Nicolaus Copernicus University, Toruń, Poland; ^2^ Department of Geriatrics, Division of Biochemistry and Biogerontology, Collegium Medicum in Bydgoszcz, Nicolaus Copernicus University, Toruń, Poland; ^3^ The Mazovian Academy, Faculty of Health Sciences, Płock, Poland

**Keywords:** β-blockers, heart failure, heart arrhythmia, COVID-19, SARS-CoV-2, propranolol

## Abstract

β-blockers is a vast group of antiarrhythmic drugs which differ in their pharmacokinetic and chemical properties. Some of them block β-adrenergic receptors selectively while the others work non-selectively. Consequently, they reduce the influence of the sympathetic nervous system on the heart, acting negatively inotropic, chronotropic, bathmotropic and dromotropic. Although they have been present in medicine since the beginning of the 1960s, they still play a crucial role in the treatment of cardiac arrhythmias. They are also first-line group of drugs used to control the ventricular rate in patients with the most common arrhythmia–atrial fibrillation. Previous reports indicate that infection with SARS-CoV-2 virus may constitute an additional risk factor for arrhythmia. Due to the aging of the population in developed countries and the increase in the number of patients with cardiac burden, the number of people suffering from cardiac arrhythmias will increase in the upcoming years. As a result the role of above-mentioned beta-blockers will remain significant. Particularly noteworthy is propranolol–the oldest beta adrenergic antagonist, which in recent years has found additional applications due to its unique properties. In this article, we reviewed the accessible literature and summarized the current guidelines on the use of beta-blockers in the treatment of cardiac arrhythmias.

## Introduction

Beta-blockers are well-established in the treatment of arrhythmias. Atrial fibrillation (AF) is the most common arrhythmia in clinical practice. It is estimated to affect 1%–4% of the population (Algalarrondo and Extramiana, 2020). The incidence of AF has increased by 33% in the last 20 years (Lippi et al., 2021). In the United States, at least 3 million to 6 million people suffer from AF (Kornej et al., 2020). It is predicted that the absolute burden of AF may increase by as much as >60% by 2050 (Lippi et al., 2021). One of the reasons for this phenomenon is the aging of the population. However, the risk of arrhythmia is not only increased in old age, but also in case of male patients with traditional cardiac risk factors, chronic kidney disease, and heart failure. Incidental arrhythmias occur with a frequency of 0.5% per year–similar to strokes or myocardial infarctions (Khurshid et al., 2018). Athletes are also a group at risk of more frequent arrhythmias (Mont et al., 2009). Intensive practicing of sports, especially endurance sports, increases the risk of AF (Abdulla and Nielsen, 2009). There are probably several mechanisms in the pathogenesis of arrhythmia in athletes–atrial ectopic disorders, increased vagal tone, chronic systemic inflammation, and collagen imbalance (Mont et al., 2009). It is known that sports training triggers the reconstruction of the heart. In a study which analyzed the echocardiographic structure of the heart of athletes, it was observed that exercise-induced heart remodeling is shifted towards standard geometry in sprinters, while in long-distance runners concentric remodeling and hypertrophy was described (Kusy et al., 2021). Another study found that long-distance runners who experienced arrhythmia or ST-segment alteration during exercise testing had a longer training history and total exercise time than those without ECG changes (Kim et al., 2021). On the other hand, it should be remembered that regular, light or moderate physical activity improves the health of the cardiovascular system and reduces the incidence of AF (Elliott et al., 2018).

Currently, the role of the SARS CoV-2 virus and its impact on the work of the heart cannot be overlooked. Among patients hospitalized due to COVID-19, an arrhythmia incident was reported in case of 7.9% of patients in New York hospitals and 16.7% of patients in Wuhan hospitals (Goyal et al., 2020; Wang et al., 2020). In contrast, as regards patients who stayed in intensive care units, the incidence of cardiac arrhythmias was as high as 44% (Wang et al., 2020). It is important to mention that 6% of COVID-19 patients also developed life-threatening arrhythmias such as ventricular tachycardia (VT) or ventricular fibrillation (VF) (Guo et al., 2020). Although this topic is still the subject of research by scientists, the current evidence suggests that the development of SARS CoV-2 infection may cause permanent damage to the myocardium and thus predispose to arrhythmias and heart failure in the future (Varma et al., 2020). However, not only the SARS-CoV-2 virus has contributed to the increase in the number of arrhythmias in recent years. Political situations can also be a factor causing heart disorders–it was noted that during the 2016 United States presidential election there was a significant increase in the incidence of cardiac arrhythmias (Rosman et al., 2021). Anger or stress may be triggered by attacks of AF but beta-blockers weaken this unfavorable physiological reaction (Lampert et al., 2019).

Based on the American Heart Association/American College of Cardiology (AHA/ACC) and European Society of Cardiology (ESC) guidelines and taking into account changes in the electrocardiographic record, we can distinguish supraventricular and ventricular arrhythmias. The division according to clinical symptoms is also important–cardiac arrhythmias may be asymptomatic, symptomatic or may only manifest themselves in the form of complications, including sudden cardiac death (SCD). The selection of the optimal treatment depends on many factors, such as the type of disorders, their etiology and the clinical picture. It is important that beta-blockers are effective not only in the treatment of cardiac arrhythmias, but also in heart failure, being well-established alongside drugs such as ACEI, aldosterone antagonists, alongside with the new, and more promising drug called levosimedan (Tycińska et al., 2020; Grześk et al., 2022). It should be remembered that regardless of whether it is an acute condition or chronic treatment–the basic group of drugs used in the treatment of arrhythmias are β-blockers–commonly known as beta blockers.

## Theoretical basis for the efficacy of beta-blockers in cardiac arrhythmias

Normal heart function depends on the presence of two separate types of cells–the cells forming the heart’s conduction system and the cardiomyocytes that perform a mechanical function. Both types of cells are dependent on each other and only the correct operation of both types of cells determines the proper functioning of the heart muscle. The synchronous interaction between these two properties is complex, precise and relatively permanent, and among other things, its disturbance may result in the occurrence of cardiac arrhythmias. The main center of the cardiac conduction system is the sinoatrial node, which imposes the rhythm on other centers in the heart. There are at least several mechanisms responsible for the development of cardiac arrhythmias. Among the basic ones, several mechanisms may be distinguished: impulse formation disorders, impulse conduction disorders and the phenomenon of circulating excitation (reentry). Behind the occurrence of tachycardia disorders is excessive stimulation of the sympathetic nervous system. The sympathetic nervous system acts on the heart through the α and β receptors. The effect of stimulation of these receptors is the intracellular influx of Ca2 + ions through voltage-dependent membrane channels. There are mainly β1 receptors in the heart, the stimulation of which increases the rhythm frequency, the speed of atrioventricular conduction and increases the contractility of the atria and ventricles (Brodde, 1993; Brodde and Michel, 1999; Grandi and Ripplinger, 2019).

β-blockers belong to the second group of antiarrhythmic drugs in the Williams classification. They work by blocking β receptors, either selectively or non-selectively. The effect of the cardiovascular system on the blockade of β-adrenergic receptors depends on the level of sympathetic tone. Their antiarrhythmic effect is the result of a decrease in the activity and speed of conduction of the stimulatory tissue and contractility of the heart muscle by inhibiting the effect of catecholamines on beta adrenergic receptors, which reduces the concentration of Ca2 + ions in the cytoplasm. The main features of β-blockers are due to the reduced stimulation of the sympathetic nervous system: reduced automatism, reduced myocardial contractility, and reduced secretion of renin from the glomerular apparatus in the renal cortex. It can be briefly stated that β-blockers have an inotropic, chronotropic, bathmotropic and negative dromotropic effect on the heart (CIBIS-II, 1999; MERIT-HF, 1999; Bristow, 2000; Brown et al., 2008; Grandi and Ripplinger, 2019).

An additional feature characterizing some of the β-blockers is intrinsic sympathomimetic activity (ISA). It is the ability to partially stimulate the β-adrenergic receptor despite its blocking. As a result, a negative inotropic effect is exerted with a much weaker negative chronotropic effect, which is of particular importance for heart failure (Pathak and Mrabeti, 2021). In this disease entity, despite the well-established position of NT-proBNP as a diagnostic and prognostic marker, new, cheaper markers such as procalcitonin, catestatin or RDW (red cell distribution width) are still being searched for, which can also be used as a predictive factor in this disease entity (Wołowiec et al., 2016; Banach et al., 2018). In addition, ISA β-blockers do not adversely affect plasma lipoproteins during long-term treatment (Jaillon, 1990).

Research indicates the presence of intracellular β1-adrenoceptors in myocytes, which, by generating a local signal, may be involved in accelerating Ca2 + uptake (Shannon et al., 2022). Beta-blockers differ in their permeability across cell membranes. Beta-blockers that cross the membrane, propranolol and carvedilol, can block intracellular activation of β1-adrenoceptors. On the other hand, sotalol and atenolol are membrane-impermeant beta-blockers, which do not block intracellular β1-adrenoceptors (Wang et al., 2021, Wang et al., 2022).

Beta-blockers may also have an additional effect–on the basis of studies analyzing the use of beta-blockers in ischemic heart disease or liver diseases, it was found that they lower the concentration of inflammatory markers such as C-reactive protein or procalcitonin (Jenkins et al., 2002; Jachs et al., 2021).

Recently, the subject of interest of scientists and clinicians is the relationship of beta-blockers with the release of nitric oxide (NO) from vascular endothelial cells and the consequences associated therewith. NO is an endogenous gas messenger stimulating guanylate cyclase through intraepithelial nitric oxide synthase (NOS-3), which increases the production of cGMP and, as a result, vasodilation. Nitric oxide synthase is an enzyme that catalyzes the synthesis of NO in two different steps–the first is the oxidation of L-arginine to Nω-hydroxy-L-arginine; then the substrate under the influence of NOS and oxygen is broken down into L-citrulline, which is accompanied by the release of NO from the vascular endothelial cells (Stuehr, 1999; Borovac et al., 2019; Nowaczyk et al., 2021). The release of NO contributes to the dilation of blood vessels, reduction of the inflammatory reaction in the vessels, vascular smooth muscle proliferation, platelet aggregation and the amount of tissue factors (Radomski et al., 1987; Vallance et al., 1989; Eduardo Toblli et al., 2012). Both nebivolol and carvedilol, due to their antioxidant properties, extend the action of nitric oxide synthase (NOS) by reducing asymmetric dimethylarginine (AMDA) and thus increase the bioavailability of NO (Garbin et al., 2007; Alfieri et al., 2008; Vanhoutte and Gao, 2013). However, in a study on the cavernous bodies of rats by Dogru et al. (2010), nebivolol significantly exceeded carvedilol and metoprolol in terms of the amount of NO released. According to the results of the study, this significant difference may result in the additional generation route of NO through the interaction of nebivolol with the estrogen receptor. It has been shown that third-generation beta-blockers improve FMD (flow-mediated dilation) values much better than second-generation beta-blockers (Peller et al., 2015). FMD is a method of assessing endothelial function–this method measures the diameter of the brachial artery before and after endothelial vasodilation induced by transient ischemia. It has been proven that endothelial dysfunction is a risk factor for atherosclerosis and thus for cardiovascular stress. Compared to other beta-blockers, nebivolol also has a beneficial effect on the lipid profile, which makes it a reasonable choice for patients with metabolic syndrome (Eduardo Toblli et al., 2012). It is also worth noting that nebivolol and carvedilol do not reduce the stroke volume of the heart, which distinguishes them from other beta-blockers. Moreover, they maintain the function of the left ventricle, thereby maintaining stroke volume, cardiac output, and maintaining cardiac chronotropism during exercise (Eduardo Toblli et al., 2012). As it turns out, modulation of the guanylate cyclase pathway may be a key therapeutic option in case of patients with cardiovascular diseases (Grześk and Nowaczyk, 2021). It also turns out that there are alternative ways of stimulating adenylate cyclase, independent of nitric oxide (Grześk and Nowaczyk, 2021). It is important to note that there are two cyclases–membrane-bound guanylate cyclase (mGC) and intracellular cyclase (sGC). Agonists for mGC are peptides, such as natriuretic peptides, and for sGC, gas mediators, such as nitric oxide and carbon monoxide (Grześk and Nowaczyk, 2021). Nitric oxide is one of the first endogenous gas transmitters, discovered in 1998. The Nobel Prize in physiology or medicine was awarded for the discovery of NO, which highlights its potential role and hopes associated with it–it seems to be a promising point for further research, especially in the field of cardiovascular diseases (Nowaczyk et al., 2021).

## Pharmacokinetic parameters of beta-blockers

One of the most important parameters differentiating individual β-adrenolytics are the pharmacokinetic properties. Pharmacokinetics is the science of describing, under the acronym LADME, the processes of liberation, absorption, distribution, metabolism, and excretion of a drug. The use of pharmacokinetics in pharmacotherapy allows the correct dose levels and length of the dose intervals to be determined. The absorption process is influenced, among others, by: the degree of ionization, solubility in the lipid layer of the biological membrane [characterized by the logarithm of the n-octanol/water partition coefficient (logP)], the size of the drug molecule and technological factors such as the drug formulation method or the auxiliary substances that were used (Ruiz-Garcia et al., 2008).

### pKa/pKb

More ionized substances are absorbed slower and to a lesser extent. The way and intensity with which a substance passes through the membrane is a function of the dissociation constant (pKa), characteristic of a given chemical compound, and the pH of the environment in which the compound is presently located. The pKa value corresponds to the pH value at which the number of ionized molecules of a given compound is equal to the number of non-ionized molecules (Manallack, 2007). Changes in pH affect the degree of ionization of compounds, which may change the degree of absorption of a given substance. Alkalizing the environment will increase the degree of ionization of weak acids, while after acidification, the degree of ionization of weak bases will increase. Therefore, substances with weak acid properties are better absorbed from a more acidic environment, and substances with a weak base character from an alkaline environment.

### pKi

**GRAPH 1:**
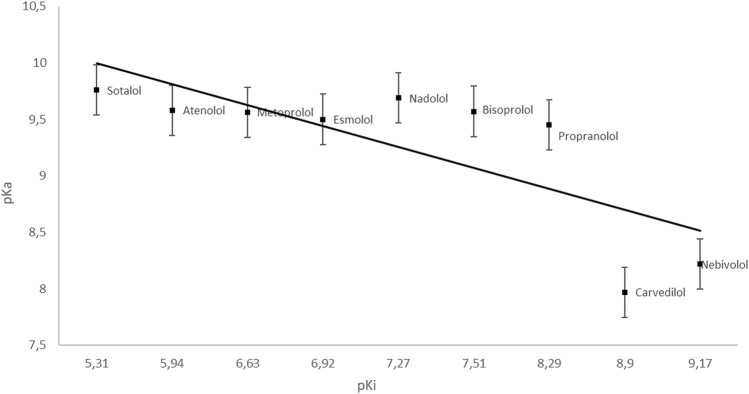
Dependence of pKa on pKi (esmolol | Ligand Activity Charts | IUPHAR/BPS Guide to PHARMACOLOGY; PubChem; Lowe et al., 2002).

**GRAPH 2:**
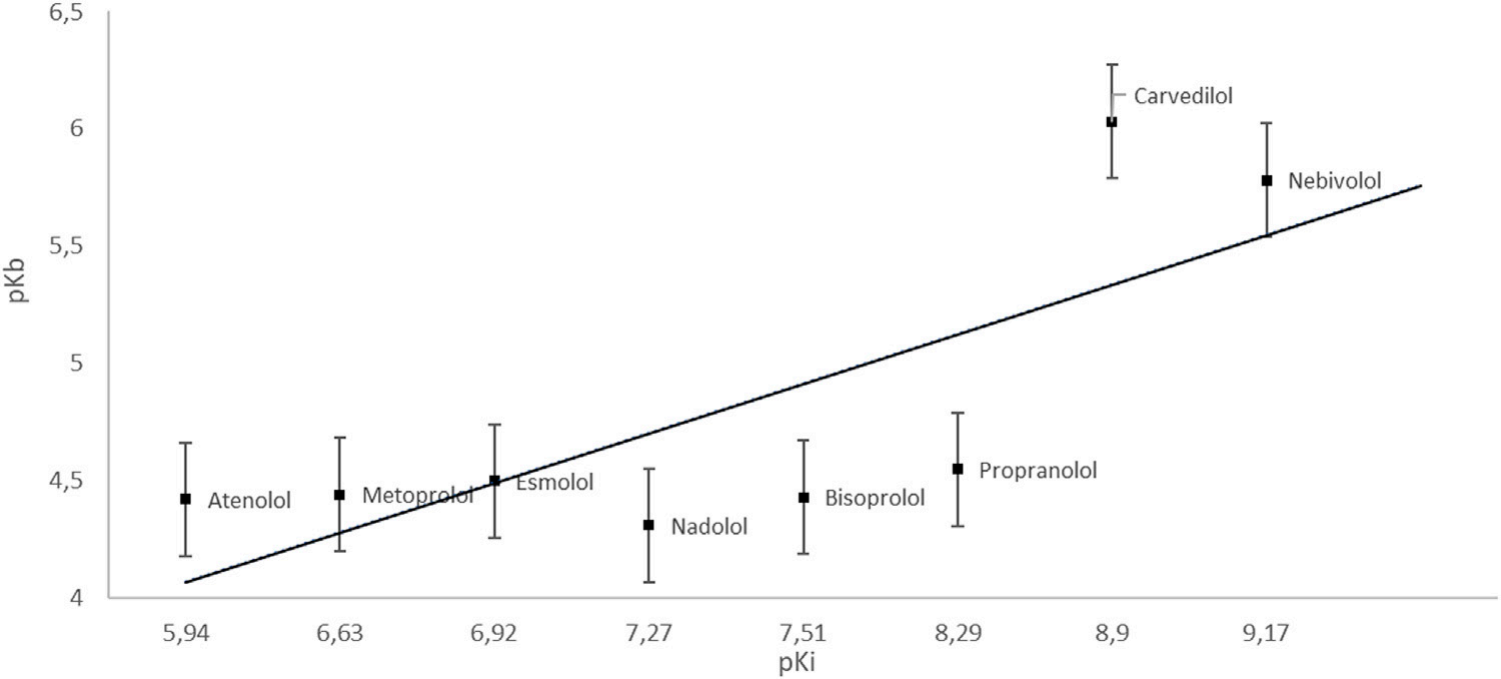
Dependence of pKb on pKi (esmolol | Ligand Activity Charts | IUPHAR/BPS Guide to PHARMACOLOGY; PubChem; Lowe et al., 2002).

Many drugs achieve their biological effect by inhibiting enzymes. To understand the mechanism of action of these drugs as enzyme inhibitors, the relationship between drug concentration and the rate of reaction performed by an isolated enzyme is studied. To describe the degree of inhibition of the enzymatic reaction, various parameters are used, including the inhibition constant–pKi, which is the dissociation constant of the enzyme-inhibitor complex reaction and the inverse of the affinity of the inhibitor to the enzyme (Yung-Chi and Prusoff, 1973) (Graph 1, Graph 2).

### logP

**GRAPH 3:**
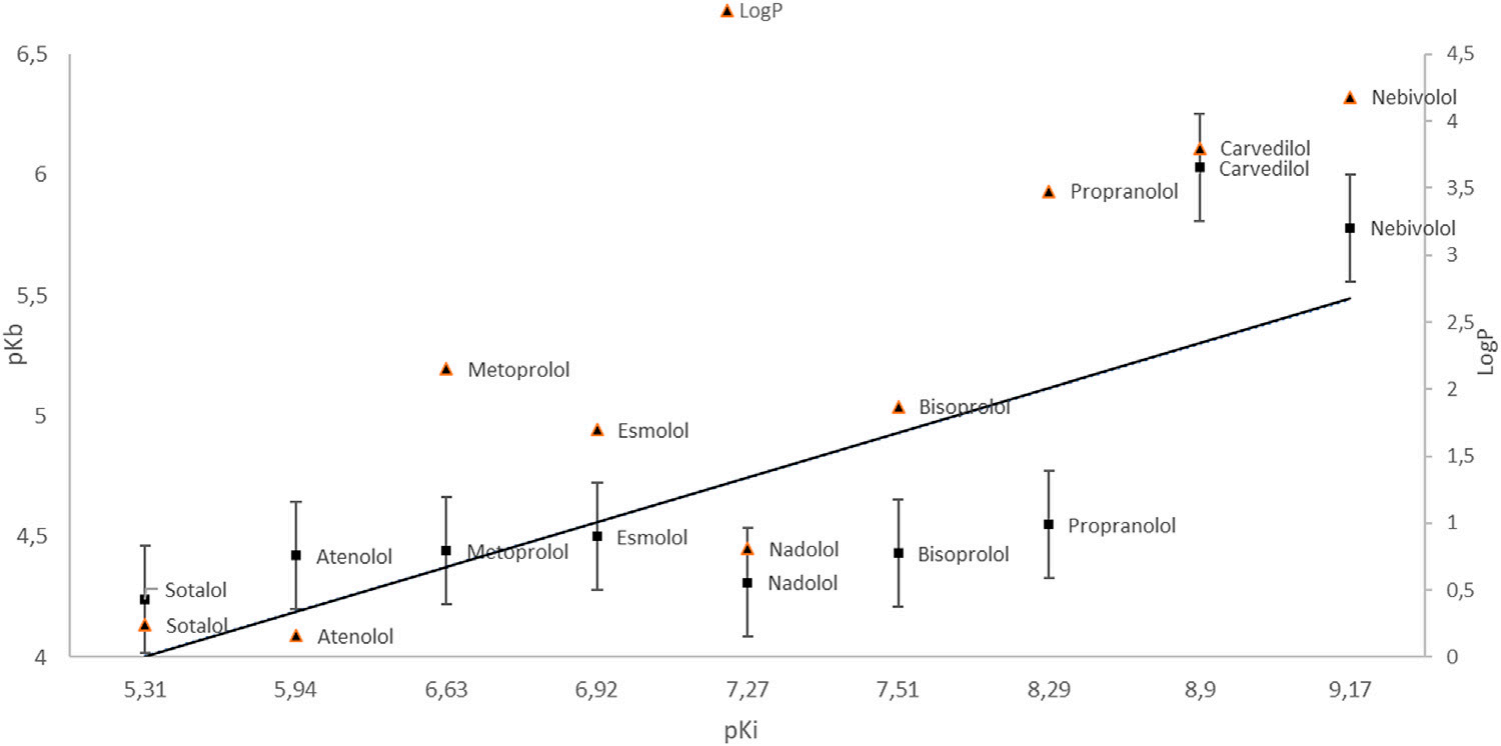
Dependence of pKb on pKi. LogP value (esmolol | Ligand Activity Charts | IUPHAR/BPS Guide to PHARMACOLOGY; PubChem; Lowe et al., 2002).

A measure of the hydrophobic-hydrophilic properties of a given compound is lipophilicity, represented by the partition coefficient P or its logP logarithm. It is a physicochemical parameter that determines transport to the site of action of the substance. The more polar a given component is, the higher the logP value and the lower the ability to penetrate lipid membranes (Arnott and Planey, 2012). However, an excessive increase in lipophilicity increases the affinity to membrane lipids and hinders the transport of compound molecules through the aqueous phase (Rudraraju et al., 2014). At extremely high logP values, the solvation interactions cause the substance to be retained in the lipophilic phase. Additionally, hydrophobic drugs are more prone to metabolic processes and elimination from the body. The aim is to select medicinal substances with optimal hydrophobic-hydrophilic properties and a partition coefficient (logP) (Arnott and Planey, 2012; Rudraraju et al., 2014). It is assumed that compounds with logP> 3 are characterized by high lipophilicity and high bioaccumulation potential (Graph 3, Table 1).

**TABLE 1:** Characteristics of individual β-blockers (Murakami et al., 2005; Gaciong and Narkiewicz, 2008; Maurer, 2010).

Drug	Cardioselectivity	ISA	Lipophilicity	t1/2 (h)	Drug excretion
Atenolol	++	−	−	6–9	renal
Bisoprolol	++	−	++	9–12	renal/hepatic
Carvedilol	−	−	++	7–10	hepatic
Esmolol	+	−	−	0.15	blood esterases
Landiolol	++	−	−	0.06	renal
Metoprolol	++	−	+++	3–4	renal
Nadolol	−	−	−	12–24	renal
Nebivolol	++	−	+	10	renal/hepatic
Propranolol	−	−	+++	3–4	hepatic
Sotalol	−	−	−	12	renal

ISA, intrinsic sympathomimetic activity; t1/2, terminal disposition half-life.

## Studies justifying the use of β-blockers in cardiac arrhythmias

Back in the early 1960s, James Black developed the first β-blocker drug, propranolol, which has been shown to have a “sedative” effect on the heart. In 1988 he was awarded the Nobel Prize in Physiology and Medicine (Stapleton, 1997). Thus, he began a wave of research on new drugs that block beta receptors. There have been many studies showing the relationship between heart rate and the risk of sudden cardiac events. One of them is the CORIDS study published in 2000, which observed that the relative risk of all-cause mortality increased with increasing heart rate (Kristal-Boneh et al., 2000). In a study conducted on people aged >70 years, it was shown that increased heart rate is associated with increased mortality in older women–however, increased heart rate was not a predictor of mortality in older men (Perk et al., 2003). In the randomized AFFIRM study published in 2004, it was shown that β-blockers were the most effective in ensuring adequate heart rhythm control in patients with AF (Olshansky et al., 2004). The VALIANT study published in 2008 showed that in patients after myocardial infarction (post-MI patients) with sustained VT/VF, treatment with a beta-blocker within the first 24 h was associated with reduced early mortality (Piccini et al., 2008). In the MADIT-II study, patients with implantable cardioverter-defibrillator (ICD) treated with the highest doses of beta-blockers experienced a significant reduction in the incidence of recurrent VT/VF requiring ICD intervention compared to patients who did not receive beta-blockers (Priori et al., 2015). These are examples selected from numerous studies showing that β-blockers are effective in treating arrhythmias. This is evidenced by the fact that β-blockers are widely used and have been included in both European and American guidelines for supraventricular and ventricular arrhythmias. There are also other benefits from the use of this group of drugs–it was noted that in hospitalized patients in severe clinical condition, treatment with beta-blockers reduced long-term mortality (Heliste et al., 2022). Also in patients with sepsis, prior exposure to beta-blockers has been associated with reduced mortality (Tan et al., 2019).

When choosing the right treatment, it is also important to pay attention to interaction - not only with drugs but also with food. A flagship example of drug and drug interactions with food are drugs from the VKA group (vitamin K antagonist). In recent studies it has been shown that the effectiveness of therapy with drugs from the group DOAC/NOAC (direct oral anticoagulants/non-Vitamin K antagonist oral anticoagulants) also depends on the type of food consumed (Grześk et al., 2021). In the case of beta-blockers, we can also observe interactions with food. For instance, caffeinated foods and drinks (such as coffee, some sodas, and soft drinks and bars) can slow down the body’s metabolism of beta-blockers, which may cause the drug to remain active for an extended period of time (Belayneh and Molla, 2020).

## Classification of cardiac arrhythmias

The classification of cardiac arrhythmias usually distinguishes two groups of arrhythmias: supraventricular and ventricular arrhythmias.

### Supraventricular arrhythmias

Traditionally, the term supraventricular tachycardia (SVT) refers to a tachycardia that originates in the His bundle or upstream structures. The traditional classification of arrhythmias classifies all types of tachycardia as SVT, excluding atrial fibrillation and ventricular tachycardia. The conventional classification of supraventricular tachycardia includes: atrial tachycardia, AV junctional tachycardia, and atrioventricular recurrent tachycardia (AVRT) (Brugada et al., 2020) (Table 2).

**TABLE 2:** Supraventricular tachycardia (SVT)–classification (Brugada et al., 2020).

Atrial tachycardias (AT)
Sinus tachycardia
• Physiological sinus tachycardia
• Inappropriate sinus tachycardia
• Sinus nodal re-entrant tachycardia
Focal AT
Multifocal AT
MRAT
• Cavotricuspid isthmus-dependent MRAT
- Typical atrial flutter, counter-clockwise (common) or clockwise (reverse)
- Other cavotricuspid isthmus-dependent MRAT
• Non-cavotricuspid isthmus-dependent MRAT
AF
AV junctional tachycardias
Atrioventricular nodal re-entrant tachycardia (AVNRT)
• Typical
• Atypical
Non-re-entrant junctional tachycardia
• JET (junctional ectopic or focal junctional tachycardia)
• Other non-re-entrant variants
Atrioventricular re-entrant tachycardia (AVRT)
• Orthodromic (including PJRT)
• Antidromic (with retrograde conduction through the AVN or, rarely, over another pathway)

AF, atrial fibrillation; AT, atrial tachycardia; AV, atrioventricular; AVN, atrioventricular node; JET, junctional ectopic tachycardia; RA, right atrial; LA, left atrial; MRAT = macro−re-entrant atrial tachycardia; PJRT, permanent junctional reciprocating tachycardia; RA, right atrial.

According to the literature on the subject, the division into narrow-complex tachycardia (QRS complexes ≤120 ms) and wide-complex tachycardia (QRS complexes lasting> 120 ms) is used. However, in clinical practice, supraventricular tachycardia may be manifested as narrow or wide QRS complex tachycardias, mostly occurring as regular rhythms.

### Atrial fibrillation (AF)

The traditional classification of atrial fibrillation distinguishes five forms of AF based on duration, clinical presentation, and spontaneous ending of AF episodes (Table 3). In the event of presence of several forms of AF (the presence of both paroxysmal and persistent AF), the diagnosis should be made in order to determine which form occurs more frequently (Hindricks et al., 2021). Atrial fibrillation can be classified according to the degree of limitation in daily physical activity–as used by the EHRA (Wynn et al., 2014) (Table 4).

**TABLE 3:** Atrial fibrillation–classification (Hindricks et al., 2021).

AF pattern	Definicja
First diagnosed	AF not diagnosed before, irrespective of its duration or the presence/severity of AF-related symptoms
Paroxysmal	AF that terminates spontaneously or with intervention within 7 days of onset.
Persistent	AF that is continuously sustained beyond 7 days, including episodes terminated by cardioversion (drugs or electrical cardioversion) after ≥7 days
Long-standing persistent	Continuous AF of >12 months’ duration when decided to adopt a rhythm control strategy
Permanent	AF that is accepted by the patient and physician, and no further attempts to restore/maintain sinus rhythm will be undertaken. Permanent AF represents a therapeutic attitude of the patient and physician rather than an inherent pathophysiological attribute of AF, and the term should not be used in the context of a rhythm control strategy with antiarrhythmic drug therapy or AF ablation. Should a rhythm control strategy be adopted, the arrhythmia would be re-classified as ‘long-standing persistent AF’

**TABLE 4:** EHRA (European Heart Rhythm Association) symptom scale (Hindricks et al., 2021).

Score	Symptoms	Description
1	None	AF does not cause any symptoms
2a	Mild	Normal daily activity not affected by symptoms related to AF
2b	Moderate	Normal daily activity not affected by symptoms related to AF, but patient troubled by symptoms
3	Severe	Normal daily activity affected by symptoms related to AF
4	Disabling	Normal daily activity discontinued

AF, atrial fibrillation.

### Ventricular arrhythmias

Under normal conditions, excitation begins in the sinus node, which is located in the right atrium. In contrast, in ventricular arrhythmias, depolarization is generated by the medium located in the right or left ventricle. The most common causes of ventricular arrhythmias include: coronary heart disease and myocardial infarction, heart defects (including congenital defects), cardiomyopathies, genetic and congenital diseases. The basic classification of ventricular arrhythmias is based on electrocardiographic changes. On this basis one may distinguish: non-sustained ventricular tachycardia (nsVT), sustained ventricular tachycardia (sVT), polymorphic ventricular torsade de pointes, ventricular flutter (VFl), ventricular fibrillation (VF). Another classification of ventricular arrhythmias applicable in clinical practice is the classification by clinical symptoms. On the basis of the clinical picture, there are haemodynamically stable disorders, haemodynamic unstable disorders, sudden cardiac arrest and sudden cardiac death (Lerma and Glass, 2016). Ventricular arrhythmias in a patient without structural or ischemic heart disease should raise suspicions of hereditary arrhythmias (Laksman et al., 2019).

## Practical aspects of β-blockers therapy in treatment of cardiac arrhythmias

### The use of β-blockers in supraventricular tachycardia (SVT)

#### The acute management of arrhythmia of unknown mechanism

In hemodynamically stable patients with a regular narrow–QRS complex tachycardias, when vagal maneuvers or adenosine are ineffective, the administration of intravenous β-blockers (ex. esmolol or metoprolol) may be beneficial (Table 5). These drugs however, prove to be more effective in reducing the heart rate than in terminating the arrhythmias (Amsterdam et al., 1991). It should be emphasised, that they can only be used in hemodynamically stable patients and moreover, they are contraindicated in the presence of decompensated heart failure. β-blockers can also be used in regular wide–QRS complex tachycardias, but only in case of SVT with aberrant conduction. When the mechanism of arrhythmia is unknown, it should be treated and managed as if it was a ventricular tachycardia (VT). Irregular SVTs are usually a manifestation of AF. The use of β-blockers in AF will be discussed further in this article.

**TABLE 5:** Dosage of beta-blockers in the treatment of SVT (Page et al., 2016; Brugada et al., 2020).

Beta-blocker	Acute therapy i.v.	Chronic therapy p.o.	Guidelines (ESC, ACC/AHA)
Atenolol	—	25–50 mg once a day (maks. 100 mg/d)	AHA
			ESC–not recommended for pregnant patients
Esmolol	0.5 mg/kg i.v. (bolus) or 0.05–0.3 mg/kg/min (infusion)	—	ESC
Metoprolol tartrate	2.5–5 mg i.v. (bolus lasting 2 min), can be repeated - bolus up to 3 doses over 10 min	25–200 mg twice a day	ESC, AHA
Metoprolol XL (metoprolol succinate)	—	50–400 once a day	ESC, AHA
Nadolol	—	40–320 mg once a day	AHA
Propranolol	1 mg i.v. (a bolus lasting 1 minute), can be repeated up to 3 times at 2-min intervals	30–160 mg in divided dose or in single doses in the case of long-acting preparations	AHA
			ESC
Sotalol	—	40–160 mg twice a day	AHA–Sotalol may be appropriate for the ongoing treatment of patients with symptomatic SVT who are ineligible for ablation or prefer not to undergo ablation
			ESC–Sotalol is not recommended as a first-line antiarrhythmic drug because its administration is associated with an increased risk of proarrhythmia and increased mortality

ESC, European Society of Cardiology; AHA/ACC, American Heart Association/American College of Cardiology.

#### Supraventricular arrhythmias

β-blockers may be considered in symptomatic patients with inappropriate sinus tachycardia unresponsive to lifestyle modifying strategies (Ptaszynski et al., 2013b; Olshansky and Sullivan, 2019). However, the combination of a β-blocker with ivabradine seems to be a more efficient treatment, compared to each of these drugs used in monotherapy (Ptaszynski et al., 2013a). In case of patients with postural orthostatic tachycardia syndrome (POTS), when non–pharmacological treatment failed, a low dose of non-selective β-blocker, midodrine or pyridostigmine should be taken into consideration (Fu et al., 2011; Kanjwal et al., 2011). Moreover, low dose of propranolol proved to decrease tachycardia, palpitations and attenuate the acute symptoms in patients with POTS (Raj et al., 2009). In acute management of focal atrial tachycardia (AT) in hemodynamically stable patients, without decompensated heart failure, the use of β-blockers may be considered, if treatment with adenosine is unsuccessful. In long-term treatment however, β-blockers should be taken into account in patients without ischaemic or structural heart disease, when the ablation is either not desirable nor feasible (Chen et al., 1994). If the current treatment has been unsuccessful, the combination of ivabradine and a β-blocker may be the next therapeutical option (Meles et al., 2015). In acute management of multifocal AT β-blockers or the non-dihydropyridine calcium channel blockers are used, when the initial treatment of the primary condition failed. However, the research have shown, that metoprolol appears to be more effective than verapamil in treating multifocal atrial tachycardia (Arsura et al., 1988). In long-term treatment selective β-blockers should be considered in patients with a recurrent and symptomatic multifocal AT (Hazard and Burnett, 1987). In case of hemodynamically stable patients with macro-reentrant atrial tachycardia (MRAT) intravenous administration of β-blockers should be taken into consideration in acute management to control and decrease the ventricular rhythm. In long-term therapy of patients with MRAT and without a heart failure, β-blockers should be considered, when the ablation is either contraindicated or not feasible (Platia et al., 1989).

#### Atrioventricular junction tachycardia

A β-blocker (propranolol) in combination with diltiazem should be taken into account in acute management of atrioventricular nodal reentry tachycardia (AVNRT). The research have proven, that it may lead to termination of arrhythmia and conversion to sinus rhythm in majority of patients (Yeh et al., 1985). Nevertheless, it is crucial to be mindful of the possible adverse effects, that may occur as a result of the interaction between these two drugs. Regarding the long-term treatment, β-blockers can only be used, if the ablation is not desirable or feasible. In treatment of junctional ectopic tachycardia (JET) propranolol can be used in acute management as well as in long-term therapy (Brugada et al., 2020).

#### Accessory pathway-mediated reentrant tachycardias (atrioventricular)

In acute management of patients with AVRT, β-blockers can be a therapeutic option, in case vagal manoeuvres and adenosine fail. In long-term treatment β-blockers should be taken into consideration, if there is no signs of preexcitation in resting ECG and when the ablation is either not desirable or feasible (Brugada et al., 2020). β-blockers should be avoided in patients with pre-excited AF, because of potential side effects in this group.

## The use of β-blockers in ventricular rate control in patients with AF

β-blockers remain one of the main rate-control agents in patients with AF and in majority of patients they are the first-line therapy (Table 6). In contrary to verapamil and diltiazem, they can be administered to patients with heart failure, which speaks in their favor. Moreover, β-blockers are preferred to cardiac glycosides, because of their ability to control the increased sympathetic tone and slow the ventricular rate during physical exercise (van Gelder et al., 2016). β-blockers are also recommended in prevention of postoperative AF in patients undergoing cardiac surgeries (Hindricks et al., 2021).

**TABLE 6:** Dosage of beta-blockers in the treatment of AF (January et al., 2014; Hindricks et al., 2021).

Beta-blocker	Acute therapy i.v.	Chronic therapy p.o.	Guidelines (ESC, ACC/AHA)
Atenolol	—	25–100 mg once a day	ESC, AHA
Bisoprolol	—	1.25–20 mg once a day (ESC)	ESC, AHA
		2.5–10 mg once a day. (AHA)	
Carvedilol	—	3,125–50 mg twice a day. (ESC)	ESC, AHA
		3,125–25 mg twice a day (AHA)	
Esmolol	500 μg/kg (a bolus lasting 1 min); then 50–300 μg/kg/min	—	ESC, AHA
Landiolol	100 μg/kg i.v. (a bolus lasting 1 min), followed by 10–40 μg/kg/min	—	ESC
Metoprolol tartrate	2.5–5 mg i.v. (a bolus), up to 4 doses (up to 3 doses–AHA)	25–100 mg twice a day	ESC, AHA
Metoprolol XL (metoprolol succinate)	—	50–400 mg once a day	ESC, AHA
Nadolol	—	10–240 mg once a day	AHA
Nebivolol	—	2.5–10 mg once a day	ESC
Propranolol	1 mg i.v. (a bolus lasting 1 min); up to a maximum of 3 doses at 2-min intervals	10–40 mg 3–4 times a day	AHA

ESC, European Society of Cardiology; AHA/ACC, American Heart Association/American College of Cardiology.

Combining β-blockers and verapamil or diltiazem is associated with a risk of bradycardia and therefore requires careful heart rate monitoring, preferably by 24-h ECG. In acute management of AF for heart rate control three of the β-blockers can be administered intravenously. The following are: metoprolol tartrate, esmolol and–newly mentioned in the recommendations–landiolol. The results of the J-Land study, conducted on 200 patients, have shown, that landiolol was more efficient in controlling HR in patients with AF and LV disfunction, compared to digoxin (Nagai et al., 2013). Furthermore, the J-Land 3S study has proven, landiolol in combination with conventional sepsis therapy to be successful in treatment of sepsis-related tachyarrhythmia, compared to conventional sepsis therapy alone. In addition to that, landiolol also reduced the incidence of new-onset arrhythmia (Kakihana et al., 2020). In oral maintenance therapy the following can be used: metoprolol tartrate and metoprolol succinate, bisoprolol, atenolol, nebivolol and carvedilol. Other β-blockers, that are available, are not recommended by the ESC as a specific rate-control agents in patients with AF. In the table below, we present the β-blockers used in rate-control in AF–according to the recommendations from the ESC 2020 and AHA 2014, respectively.

## The use of β-blockers in ventricular arrhythmias (VA)

The effective management of underlying diseases and co-morbidities is fundamental for the successful treatment of ventricular arrhythmias and prevention of sudden cardiac death. By the selection of an appropriate therapy for the VA and for prevention of SCD the following factors should be taken into account: type of arrhythmia, the accompanying diseases, the risk posed by arrhythmia and the potential adverse events of such therapy. Even though β-blockers remain a relatively safe drug group, a recent study, in which a group of 34,661 patients with STEMI or NSTEMI was examined, showed that in patients with two or more risk factors for shock (such as: age >70 years, symptoms >12 h (STEMI patients), systolic blood pressure <120 mm Hg), the risk of death or shock was significantly higher in patients treated with β-blockers in acute management (Kontos et al., 2011). Nevertheless, no other antiarrhythmic agents, except for β-blockers, have proven to be efficient main therapeutic agents in management of life-threatening arrhythmias or in prevention of SCD. Furthermore, β-blockers are anti-ischemic and reduce the mortality by approximately 35% (McMurray et al., 2012). In conclusion, β-blockers are the first-line treatment in management of VA and preventing SCD (Priori et al., 2015). Sotalol is effective in management of VA and can be safely administered in patients with ischaemic heart disease (Smith et al., 2013). However, concerning its proarrhythmogenic potential, sotalol should not be used in patients with heart failure (without ICD). The administration of sotalol requires monitoring using ECG–especially in patients with low BMI or in patients with impaired renal function.

### Patients with ICD

In OPTIC trial the researchers wanted to determine, whether the use of beta-blockers alone, sotalol alone or beta-blockers with amiodarone prevents the ICD shocks (Connolly et al., 2006). The combination of amiodarone and a beta-blocker significantly reduced the number of ICD shocks, compared to beta-blocker or sotalol alone (Connolly et al., 2006). However effective, it must be taken into account, that this drug combination has an increased risk of drug-related adverse effects, which may lead to discontinuation of treatment in a significant number of patients (Hohnloser et al., 2006). According to ESC, sotalol can be used, unless there are contraindications.

### Treatment of VA and prevention of SCD in patients with ischaemic heart disease

The use of a β-blocker is recommended in management of patients with acute coronary syndrome without VA, in order to prevent ventricular fibrillation (VF) (Priori et al., 2015). In case when VF has occurred in association with ACS, an early (preferably intravenous) administration of a β-blocker can prevent recurrent events of arrhythmia (Huikuri et al., 2001; Piccini et al., 2008). Oral treatment with beta-blockers should be considered in all ACS patients without contraindications during the hospital stay and continued thereafter (Priori et al., 2015). In an early stage after MI, apart from an revascularization, an optimal medical treatment (including a β-blocker) is recommended. Furthermore, prevention and treatment of HF are also recommended in this patient group and remain the mainstays of prevention of SCD (Priori et al., 2015). β-blockers are essential for reducing the mortality of patients with a reduced LVEF after the MI. Although, they may also be beneficial for patients with preserved LVEF, the effect of beta-blockers on SCD has yet to be proven (Priori et al., 2015).

### Cardiomyopathies

Beta-blockers are recommended in management of patients with dilated cardiomyopathy (DCM) to decrease the risk of SCD and attenuate the progress of HF, as well as in case of recurrent VA (Priori et al., 2015). In patients with hypertrophic cardiomyopathy (HCM) with a not tolerated, sustained VT the implantation of ICD along with a treatment with beta-blockers should be considered (in order to suppress further episodes of arrhythmia). β-blockers should also be taken into consideration in management of patients with left ventricle outflow tract (LVOT) obstruction. However, in this particular group of patients, there is no evidence, that β-blockers reduce the risk of SCD (Huikuri et al., 2001). β-blockers are the first-line treatment in order to improve symptoms in patients with the arrhythmogenic right ventricle cardiomyopathy (ARVC) and frequent premature ventricular complexes (PVC) and non-sustained VT (NSVT).

### Inherited primary arrhythmia syndromes

β-blockers are also the first-line treatment in some of cardiac channelopathies. Patients with congenital long QT syndrome (LQTS) are recommended for β-blockers treatment (Priori et al., 2015). Beta-blocking agents should also be considered in patients, who are carriers of a causative LQTS mutation, but have a normal QT interval (Priori et al., 2015). Furthermore, beta-blockers are recommended in patients clinically diagnosed with catecholaminergic polymorphic ventricular tachycardia (CPVT), based on the presence of documented spontaneous or stress-induced VAs. In this group of patients, β-blockers without the intrinsic sympathomimetic activity are recommended (Priori et al., 2013). Moreover, for genetically positive family members of patients with CPVT, therapy with beta-blockers should be considered, even after a negative exercise test (Priori et al., 2015).

Also, the latest, recently published guidelines from 2022 emphasize the importance of beta-blockers in the treatment of ventricular arrhythmias. For instance, they are recommended in LQTS patients to reduce risk of arrhythmic events and in all patients with a clinical diagnosis of CPVT. In patients presenting with a hemodynamically tolerated idiopathic VT, treatment with intravenous beta-blocker is also recommended. The latest guidelines invariably emphasize the use of beta-blockers in ventricular arrhythmias, cardiomyopathies and channelopathies (Zeppenfeld et al., 2022).

## β-blockers in management of arrhythmias during pregnancy

Beta-blockers are considered a relatively safe group of drugs during pregnancy. However, they may cause, among others, bradycardia or fetal hypoglycemia. In order to minimize the risk of possible complications for the fetus, the selection of the appropriate drug should be aimed at the lowest effective dose of the drug. It has been observed that the use of β-blockers by a woman in the first trimester of pregnancy is not associated with a large increase in the risk of any malformation in the fetus. Intravenous preparations are used both in patients with SVT and in AF attacks (Hindricks et al., 2021). Selective beta-blockers are also preferred in the chronic treatment of supraventricular disorders (Brugada et al., 2020). Beta-blockers are also used in ventricular arrhythmias–in patients with LQTS or catecholamine-dependent polymorphic ventricular tachycardia, and in the long-term treatment of idiopathic sustained ventricular tachycardia (Priori et al., 2015). β-1 selective blockers (e.g., metoprolol and bisoprolol) are generally safe and recommended as first line agents. The drug not recommended during pregnancy is atenolol–its use was associated with a higher frequency of fetal growth retardation (Lydakis et al., 1999). Beta-blockers can also be used to treat hypertension in pregnant women–along with methyldopa and some calcium channel blockers (Grześk et al., 2019).

## β-blockers in management of COVID-19 patients–arrhythmias and additional aspects

The current research have shown, that the SARS-CoV-2 infection is associated with a greater risk of arrhythmias (Bhatla et al., 2020). In a study conducted by Peltzer et al. (2020a), in 1,053 hospitalized patients with COVID-19, arrhythmias were identified in 25.6% of patients. At the same time, the risk of arrhythmias was significantly higher in patients hospitalized in intensive care units (ICUs) (Bhatla et al., 2020; Wang et al., 2020). In another study, German patients, who have recently recovered from COVID-19, were examined with a cardiovascular magnetic resonance (CMR), which revealed cardiac involvement in 78% of the patients and myocardial inflammation in 60% of the examined group, independently of severity and overall course of the acute illness (Puntmann et al., 2020). However, it should be emphasized, that the mechanism underlying the arrhythmias in patients with SARS CoV-2 infection is complex. Not only may they be a consequence of the viral infection itself and the myocardial inflammation caused by it, but also they can occur due to systemic inflammation, adverse effects of drugs (ex. QT-prolonging drugs), autonomic imbalance or hypoxia (Manolis et al., 2020). AF was the most prevalent (21%) arrhythmia among patients with COVID-19, for 9.6% of patients it was the first episode of AF (Manolis et al., 2020). VAs were much less common, the most prevalent among them was non-sustained VT (6.3%) (Gopinathannair et al., 2020).

A retrospective observational study conducted by Russo et al. (2020), which included data of 414 patients with COVID-19 hospitalized in ten Italian hospitals, showed that the recurrent AF, as well as just one episode of AF, were significantly associated with a subsequent incident of VT. In this study however, neither the first, nor the following episodes of AF were associated with a greater risk of ARDS or death. An incidence of VT, on the contrary, was an independent risk factor of in-hospital mortality in patients with COVID-19. On the other hand, a different study conducted by Peltzer et al. (2020b) proved that in hospitalized patients with AF or AFl the mortality was three times higher, compared to patients without arrhythmias. The current ESC guidelines for the diagnosis and management of cardiovascular disease during the COVID-19 pandemic, recommend to use beta-blockers, among other drugs, in heart rate control in AF, with the exception of patients with acute respiratory insufficiency, where considering the risk of further deterioration of lungs function, calcium channel blockers should be the treatment of choice. In hemodynamically unstable patients, after cardioversion was performed, one of the agents, that can be used is sotalol. In management of patients with COVID-19 and VAs intravenous esmolol is recommended in hemodynamically stable patients with a polymorphic VT or VF–when QTc is not prolonged. Furthermore, it esmolol administered intravenously can be used in patients with a recurrent monomorphic VT, who receive QT-prolonging antiviral medication. The authors of the ESC guidelines lay great emphasis on analysis of possible drug to drug interactions before administering any new medicament–especially antiviral agents (considering their QT-prolonging potential), antiarrhythmic or anticoagulant therapy (Task Force for the management of COVID-19 of the European Society of Cardiology, 2022).

Beta-blockers also present with various other, less known, nevertheless very promising qualities, especially in the midst of the COVID-19 pandemic. They have an anti-inflammatory effect by inhibiting the release of IL-6 and TNF-α, allowing the mitigation of the cytokines storm in the COVID-19. Moreover, beta-blocking agents decrease the release of catecholamines caused by SARS CoV-2 infection and also attenuate the development of the sympathetic storm. Furthermore, metoprolol reduces the oxidative stress as it blocks the activity of myeloperoxidase (MPO)—an enzyme, which is one of many common steps linking the development of the cytokines and sympathetic storms. Those two processes interacting together lead to lethal complications in patients with severe COVID-19 (Al-Kuraishy et al., 2021).

The results of a study conducted by Clemente-Moragón et al. (2021) appear to be especially promising. In this study metoprolol was administered intravenously to critically ill, mechanically ventilated COVID-19 patients with ARDS. In patients, who received a three-day treatment with 15 mg of i.v. metoprolol a day, a significant decrease of neutrophil content in bronchoalveolar lavage (BAL) was observed, along with a decrease of lung inflammation markers, in comparison with a control group (no metoprolol). Furthermore, patients who received metoprolol required fewer days on invasive mechanical ventilation and a significant improvement in oxygenation was observed, compared to the control group. A study by Chouchana et al. (2022) conducted using data of 8,078 hospitalized patients from 39 university hospitals in Paris, showed a lower mortality rate in patients with COVID-19 and preexisting hypertension, who received beta-blockers as hypotensive agents, which may suggest a possible protective effect of these drugs. What is known so far about the qualities of β-blockers should encourage further research in the future, as they seem promising as a possible therapeutic option for patients with COVID-19.

## Propranolol–an old drug with new properties

Having summarized the sites of beta-blockers in the guidelines of cardiac arrhythmias, it is impossible not to devote a separate chapter to propranolol–the first beta-blocker invented in the 1960s and recently experiencing its renaissance. Propranolol is a non-selective β1-and β2-adrenergic receptor antagonist. Until now, it was known as a drug used in cardiovascular diseases that lowers the heart rate and blood pressure. These properties have been known for many years. However, in recent years, it has attracted attention again due to new, potential therapeutic directions in which it could be used, including tumors and rare diseases. The pharmacodynamics of propranolol show that this drug has vasoconstrictive and anti-angiogenic effects and contributes to the induction of apoptosis in some types of cells. By blocking adrenergic receptors, propranolol has a vasoconstrictive effect–abovementioned drug causes the constriction of blood vessels, reducing blood flow that nourish the tumor, thus inhibiting tumor growth. Propranolol also reduces the level of Hypoxia-Inducible Factor (HIF), which is responsible for the regulation of VEGF–the main proangiogenic factor. As a result, the tumor-related angiogenic process is impaired. In addition, various studies have shown that blockade of β1-and β2-adrenergic receptors by propranolol can induce apoptosis in some types of cells and tumors *in vitro*, for example in the capillary endothelial cells (Sommers Smith and Smith, 2002). However, this mechanism is not fully understood. It is also important that propranolol has some lipophilic properties, thanks to which it can cross the blood-brain barrier (Olesen et al., 1978).

All these mechanisms contributed to the successful use of propranolol in the treatment of infantile hemangiomas (IH) (You et al., 2021; Cuesta et al., 2022). It has been shown that this indication is more effective than steroids, although patients treated with propranolol should be monitored due to the risk of recurrence and the possible need for re-treatment (Frongia et al., 2021; You et al., 2021). An alternative to oral treatment may be patches with propranolol (Musazzi et al., 2021). Nadolol can be an effective and safe alternative to propranolol (Pope et al., 2022). Also in the treatment of subglottic hemangiomas, propranolol and nadolol have been shown to be equally effective (Yang et al., 2021). However, in a recent study, the main role of propranolol was questioned as it was shown that atenolol can be considered the first-line treatment of infantile hemangiomas, as adverse events were less frequent in the atenolol-treated group, with similar response rates (Ji et al., 2021). Propranolol lead to reduced nighttime sleep efficiency and increased daytime sleep requirements, but overall the effects were mild (Theiler et al., 2021). There was no negative effect of propranolol on behavioral development in children (Theiler et al., 2021). The time of using propranolol may be shortened if the laser is added to the therapy (Sugimoto et al., 2021). Following the success of propranolol in the treatment of IH, propranolol has been used in clinical trials for various types of tumors and for rare diseases such as cerebral cavernous malformation (CCM).

Taking into consideration the wide and nuanced application of beta-blockers over the years, it is impossible not to mention their use in the treatment of situational anxiety, such as stage fright, fear of public speaking or performance anxiety on various fields. In a double blind placebo controlled study conducted by Brantigan et al. (1979) the administration of oral propranolol not only did eliminate the physical barriers and the dry mouth feeling caused by performance anxiety, but also helped to improve the quality of the musical performance itself. Furthermore, a very interesting randomized, double-masked, crossover study by Elman et al. (1998) showed, that 40 mg of propranolol taken 1 h before the surgery, significantly reduced the tremor and anxiety among ophthalmology residents, without causing any adverse effects neither to the surgeon, nor to the patient. However, in another study, treatment with propranolol did not substantially improve the performance of participants with public speaking anxiety, compared to placebo. Non-etheless, participants reported a reduction of speech distress and speaking anxiety following treatment (Elsey et al., 2020). In spite of the abovementioned, the systematic review and meta-analysis conducted by Steenen et al. concluded, that currently there is no sufficient evidence for the routine administration of propranolol in case of any anxiety disorders (Steenen et al., 2016). Moreover, another meta-analysis also showed a lack of evidence of satisfactory quality to determine, whether or not to use propranolol in the treatment of stage fright (Szeleszczuk and Frączkowski, 2022).

In recent years, a number of studies have been conducted on the possible use of propranolol in oncology. As it turned out, it can be useful in many areas. Propranolol in high doses inhibits the undesirable effects of cisplatin, including on histological symptoms as well as BUN and creatinine levels, which may prove that it reduces the nephrotoxicity of cisplatin (Esmaeeli et al., 2022). Additionally, it has been demonstrated that propranolol can reduce the metastasis of distant breast cancer, and it has been investigated that it is possible to combine propranolol with taxane/anthracycline-based neoadjuvant chemotherapy in patients with breast cancer (Hopson et al., 2021). Therapy with propranolol and metformin may be useful as an adjuvant therapy for both triple negative breast cancer and colorectal cancer, and as an alternative to chemoresistant colorectal cancer, providing an inexpensive alternative therapy without the accompanying toxicity (Anselmino et al., 2021). In the case of angiosarcoma, a very rare neoplasm, and doxorubicin therapy, drug resistance develops due to the high accumulation of doxorubicin in the lysosomes. Studies suggest that by targeting the lysosomal compartment, propranolol reduces lysosomal sequestration of doxorubicin while blocking the efflux of doxorubicin and increasing its total intracellular accumulation. This may reduce drug resistance, which is a significant problem in the case of doxorubicin therapy (Saha et al., 2020).

The effect of beta-blockers on breast, ovarian, colorectal and pancreatic cancer has been investigated in observational studies (Wen et al., 2021). In studies, propranolol has been shown to inhibit cell proliferation, and the combination of propranolol, docetaxel and doxorubicin in the treatment of various soft tissue sarcomas resulted in a better response to treatment, the most sensitive were angiosarcoma and liposarcoma (Porcelli et al., 2020). Non-selective beta-blockers are more effective than selective beta-blockers in inhibiting angiosarcoma cancer, but also ovarian, breast and liver cancer (Thiele et al., 2015; Watkins et al., 2015; Montoya et al., 2017). Proporanolol seems to be effective also in patients with metastatic angiosarcoma (Amaya et al., 2018). The anti-angiogenesis-inhibiting effect and the immunostimulating effect of propranolol have been suggested as an alternative anti-cancer treatment (Pasquier et al., 2016). In a study of breast cancer patients, in which propranolol was administered 7 days before breast resection, it was shown that the frequency of metastases was inversely correlated with the administration of a beta-blocker (Hiller et al., 2020). In pancreatic and prostate cancer, observational studies have shown a reduction in tumor-related mortality with beta-blocker therapy. However, in ovarian cancer, there is no benefit (Carlos-Escalante et al., 2021).

Last studies have shown that propranolol may also have beneficial effects in other diseases. In post-traumatic stress disorder (PTSD), propranolol did not show a beneficial effect on PTSD symptoms, but significantly reduced the heart rate after traumatic memory recall as compared to placebo (Raut et al., 2022). Research involving propranolol has identified potential cellular and molecular mechanisms involved in tremor reduction and identifies relevant genetic biomarkers for the drug response in essential tremor (ET). Propranolol has been found to influence the expression of genes previously associated with essential tremor and other movement disorders such as TRAPPC11 (Castonguay et al., 2022). Propranolol also proved to be effective in reducing pain associated with temporomandibular disorders in people with migraine (Tchivileva et al., 2021). In addition, administration of propranolol prior to memory reactivation disrupted the re-fixation of smoking memories in smokers. These findings show noradrenergic regulation of memory reconsolidation in humans and suggest that additional administration of propranolol may facilitate the treatment of nicotine addiction (Lin et al., 2021). Propranolol, like metformin, can reduce insulin resistance and heart remodeling, possibly by increasing β-arrestin2 signaling activity. This is an important discovery, because modulation of β-arrestin2 signaling may be a promising strategy for the treatment of insulin resistance (Ibrahim et al., 2021). The utility of propranolol has been demonstrated in at least one more disease. CCM is a collection of small blood vessels in the central nervous system that are enlarged and irregularly structured. In CCM, the capillary walls are thinner than normal, less flexible, and likely to leak. Animal studies have shown that propranolol also inhibits the development of cavernous vascular malformations through β1 adrenergic receptor antagonism (Li et al., 2021). Propranolol reduces the development of lesions and rescues the barrier function in cavernous brain defects. This study confirms the concept that propranolol can be used to reduce and stabilize vascular changes and therefore it can be suggested as a pharmaceutical drug in CCM (Oldenburg et al., 2021). Finally, it is worth adding that it was found that the toxicity of propranolol in rivers is very low and poses a very low risk to aquatic organisms (Sumpter et al., 2021).

Propranolol has undoubtedly become an object of interest in many areas of medicine–and for good reason. Propranolol is an example of an extremely valuable drug, showing numerous therapeutic properties in many areas, not only in treatment of cardiovascular diseases.

## Summary

β-blockers are one of the main groups of drugs used in management of arrhythmias. Their specific qualities and efficacy however, does not simply result from the blockade of beta-receptors, but also from many additional acting mechanisms, as each of these agents is pharmacologically diverse. Beta-blockers are widely used and have been known for decades now. Even though, 60 years has passed since the invention of propranolol, new beta-blocking agents, such as intravenously administered landiolol, are still being discovered. The pharmacochemical diversity of this group of drugs indicates a necessity for further research on therapeutic qualities and potential adverse effects of newly invented beta-blockers. The most important factors, that should be considered, while choosing the most suitable β-blocker are the type of the arrhythmia, the patient’s profile and the accompanying diseases along with appropriate guidelines. Nowadays, the COVID-19 pandemic already caused and most likely will still cause an increased number of new-onset arrhythmias. Also in this group of patients beta-blockers are widely used. Moreover, in one study the hypotensive therapy with beta-blockers in patients with COVID-19 and preexisting hypertension was associated with lower mortality. In conclusion, regardless of the type of the arrhythmia beta-blockers remain one of the most essential agents used in management of heart rhythm disorders.
